# Therapeutic effects of organic zinc on reproductive hormones, insulin resistance and *mTOR* expression, as a novel component, in a rat model of Polycystic ovary syndrome

**DOI:** 10.22038/IJBMS.2019.36004.8586

**Published:** 2020-01

**Authors:** Faeze Fazel Torshizi, Mohammad Chamani, Hamid Reza Khodaei, Ali Asghar Sadeghi, Seyed Hossein Hejazi, Reza Majidzadeh Heravi

**Affiliations:** 1 Department of Animal Science, Science and Research Branch, Islamic Azad University, Tehran, Iran; 2 Department of Animal Sciences, Islamic Azad University, Golpayegan Branch, Isfahan, Iran; 3 Skin Disease and Leishmaniasis Research Center, Department of Parasitology and Mycology, School of Medicine, Isfahan University of Medical Sciences, Isfahan, Iran; 4 Department of Animal Sciences, Ferdowsi University of Mashhad, Mashhad, Iran

**Keywords:** Insulin resistance, mTOR, Polycystic ovary syndrome, Rat, Zinc-methionine

## Abstract

**Objective(s):**

Zinc is an effective factor in the reproductive system. Insulin resistance (IR) is known as an important disorder in patients with polycystic ovary syndrome (PCOS). Mammalian target of rapamycin (*mTOR)*, which controls key cell activities, in particular, is activated in disorders such as PCOS. The present study was conducted to observe the therapeutic effects of organic zinc on IR, *mTOR* gene expression, and pathogenesis of PCOS in a rat model induced-PCOS.

**Materials and Methods:**

Experimental treatments were performed on control and treated groups, consisting of healthy controls (Control, water, and standard feed intake and daily injection of sesame oil alone), Polycystic control (PCO, injection of 4 mg/kg estradiol valerate (EV) for four weeks). Treated groups (PCO-ZM 25, PCO-ZM 75, and PCO-ZM 175) after 4 weeks of receiving EV, were daily given three levels of 25, 75, and 175 mg zinc methionine/kg BW for 15 days, respectively.

**Results:**

Injection of EV dramatically increased body and ovarian weights, levels of LH, testosterone, estradiol, triglyceride, fasting insulin, fasting glucose, HOMA-IR, IGF-1, gene expression of *mTOR*, and number of cysts (*P*<0.05). It also reduced the level of progesterone, HDL-C, and the number of antral follicles (*P*<0.05). However, by increasing zinc-methionine application especially at 175 mg/kg BW, the induction effects of EV were improved on ovarian cysts (*P*<0.05).

**Conclusion:**

Organic zinc showed beneficial effects in the EV induced PCOS rats via decreased insulin resistance and *mTOR* expression, restored the hormonal profile, and decreased the number of cysts in the ovaries.

## Introduction

Polycystic Ovary Syndrome (PCOS) is a multifactorial endocrinopathy disorder that occurs with clinical heterogenic symptoms such as hyperandrogenism, menstrual disorders, infertility, and hirsutism (1). The negative and extensive effects of this syndrome on physiology and metabolism of the body leads to its recognition as a metabolic disorder with visible anomalies such as high blood insulin, insulin resistance, abdominal obesity, and lipid-related disorders, which in the long term increase prevalence of these disorders (2). The management of PCOS is a major clinical challenge in gynecology (3). Insulin resistance (IR) is an important disorder in patients with polycystic ovary because of the association between inflammatory and insulin signaling pathways (4). IR may increase steroidogenesis and stimulates the hypothalamus to release LH in PCOS by hyperinsulinemia (5). Studies have suggested that hyperinsulinemia caused by IR and hyperandrogenism are reciprocal causes (6). Insulin resistance (IR) is an important disorder in patients with polycystic ovary because of the association between inflammatory and insulin signaling pathways (4). Higher sensitivity to androgens and enhanced androgen levels up to 70% have been reported in patients with PCOS (7). Hyperandrogenism may be interfered in hyperinsulinemia via reducing insulin production and enhancing insulin receptors (8). In patients with PCOS, insulin also stimulates the production of ovarian androgen through activating its homologous receptor and /or hypersensitivity to it (9).

The mammalian target of rapamycin, commonly known as *mTOR*, is a serine/threonine kinase. It has been shown as a metabolic signal in all forms of life and is involved in the control of different stressors, growth factors, nutrients, and hormones and plays major roles in the control of key cellular activities, such as growth, metabolism, cell proliferation, and differentiation (10). It may also be in association with the development of disorders such as PCOS and ovarian cancer (11). There is evidence showing that *mTOR* signaling plays an important role in the control of puberty onset, gonadotropin secretion, and reducing LH secretion in female rats (12). It has been reported that *mTOR* activity as a novel mitotic survival checkpoint, controls the follicular growth under *in vitro *condition (13). Studies have emphasized the importance of *mTOR* and cell signaling pathways, which are expressed and activated in a different way by steroid hormones and growth factors (10). The *mTOR* activity is promoted via insulin receptors and growth factors. Studies have shown that *mTOR* signaling pathways (*mTORC1* and *mTORC2*) may have responsibility in the increase of proliferation of ovarian follicular cells and their growth in PCOS mouse (14).

Zinc (Zn) is an essential factor in the reproductive system and is linked with many enzymes for regulating several metabolic activities in the body (15). Inorganic Zn may have lower absorption efficiency and reduce the stability of nutrients in the diet, and nowadays, amino acid chelated trace minerals, have received more attention due to their enhanced absorption and bioavailability (16). The serum concentrations of some of the trace elements, such as Zn may be reduced in patients with PCOS. Guler *et al.* have shown the mean level of serum Zn to be lower in patients with PCOS with impaired glucose tolerance compared with patients with normal glucose tolerance; however, the differences were not significant. They believed that zinc deficiency might play a major role in the pathogenesis of PCOS, which may be associated with its long-term metabolic consequences (17). The importance of Zn for the synthesis and action of insulin has been reported in healthy individuals and diabetic patients (18).

The causes of female infertility are often problems in the ovary, which are usually caused by PCOS. It has been reported that in women with PCOS, the high level of LH is due to an increase in the rate and intermittent secretion (9). It has been shown that Zn supplementation reduced testosterone levels and IR in women with PCOS (17). The role of Zn as a stimulator of *mTOR* activity has also been documented *in vitro *(19). It also directly regulates *mTOR* activity (20). Multiple drug methods have been offered for treating PCOS, but, due to the side effects of drugs, it is essential to use natural resources or supplement drugs that have the least or no side effects. According to the background research, Zn supplements have beneficial effects on some of the biochemical and reproductive parameters in patients with PCOS. On the other hand, organic zinc has better absorption than its other forms. This study was aimed to investigate the beneficial effects of organic Zn supplementation on insulin resistance, fertility, and *mTOR* gene expression as a novel component of polycystic ovary syndrome in PCOS rats.

**Table 1:** Primers used in the present study

Sequences	Fragment	Name
Forward, TGC CTT CAC AGA TAC CCA GTA C	141	*mTOR*-Rat
Reverse, AGG TAG ACC TTA AAC TCG GAC C		
Forward, AGA TGA CCC AGA TCA TGT TTG AGA C	120	*Actb*-Rat
Reverse, AGT CCA TCA CAA TGC CAG TGG		

**Table 2:** The effects of different levels of zinc-methionine supplementation on the body weight and the weight of reproductive tissues in rats by inducing polycystic ovary syndrome

Treatments				Control	Weighted Parameters
PCO-ZM175	PCO-ZM75	PCO-ZM25	PCO		
206.00±9.02^(*, +)^	199.00±12.08^(*, ++)^	226.600±6.59^(***)^	224.00±8.00^(^^**^^)^	187.2±15.12	BW (g)
0.058±0.003^(++)^	0.056±0.003^(+++)^	0.068±0.001^(^^***^^)^	0.067±0.002^(^^**^^)^	0.055±0.005	Ovaries/BW (mg/g)
0.42±0.03^(+++)^	0.41±0.04^(+++)^	0.25±0.03^(^^***^^)^	0.23±0.05^(***)^	0.40±0.006	Uteri/BW(mg/g)

The data are presented as mean±SD *, **, and ***, respectively show significant differences at the levels of *P*<0.05, *P*<0.01, and *P*<0.001, between the healthy control and PCO groups. +, ++, and +++ showed significant differences between zinc-methionine-treated PCO groups and PCO control group at the levels of *P*<0.05, *P*<0.01, and *P*<0.001, respectively. BW: body weight; Control: Healthy control group; PCO: Polycystic ovary control group; PCO-ZM 25, PCO-ZM 75 and PCO-ZM175 groups: polycystic groups treated with 25, 75, and 175 mg zinc-methionine; respectively

**Table 3:** Effects of various levels of zinc-methionine supplementation on the reproductive hormones in rats with induced polycystic ovary syndrome

Treatments				Control	Reproductive Hormones
PCO-ZM175	PCO-ZM75	PCO-ZM25	PCO		
4.56±0.38^(**, +)^	4.16±0.27^(*, ++)^	5.22±0.39^(^^***^^)^	5.25±0.49^(^^***^^)^	3.69±0.31	LH (mIU/mL)
5.32±0.34^(+++)^	5.44±0.33^(+++)^	2.11±0.29^(^^***^^)^	2.07±0.34^(^^***^^)^	5.26±0.23	FSH(mIU/mL)
0.25±0.14^(+++)^	0.31±0.17^(+++)^	0.64±0.11^(^^***^^)^	0.63±0.09^(^^***^^)^	0.17±0.05	TotalT(ng/mL)
3.96±0.24^(+++)^	3.79±0.17^(+++)^	3.01±0.31^(^^***^^)^	3.07±0.29^(^^***^^)^	4.09±0.30	P4 (ng/mL)
49.42±12.66^(*, +++)^	42.48±10.41^(**, +++)^	66.87±13.64^(^^***^^)^	66.71±12.83^(^^***^^)^	33.76±8.29	E2 (pg/mL)

The values are shown as mean±SD *, **, and ***, respectively show significant differences at the levels of *P*<0.05, P<0.01, and *P*<0.001, between healthy control group and PCO groups. +, ++, and +++ showed significant differences between zinc-methionine-treated PCO groups and PCO control group at *P*<0.05, *P*<0.01, and *P*<0.001, respectively. LH: luteinizing hormone; FSH: follicle-stimulating hormone; T: testosterone; P4: progesterone; E2: 17β -estradiol; Control: Healthy control group; PCO: Polycystic ovary control group; PCO-ZM 25, PCO-ZM 75 and PCO-ZM175 groups: polycystic groups treated with 25, 75, and 175 mg zinc-methionine; respectively

**Table 4:** The effects of various levels of zinc-methionine supplementation on lipid parameters

Treatments				Control	Lipid Parameters
PCO-ZM175	PCO-ZM75	PCO-ZM25	PCO		
90.80±11.34^(++)^	106.00±13.22^(+)^	128.20±11.34^(^^*^^)^	135.00±22.90^(^^*^^)^	98.20±16.9	TG (mg/dl)
41.20±4.49^(++)^	37.20±6.01^(+)^	29.80±6.45^(*)^	27.00±4.39^(^^*^^)^	36.80±7.25	HDL- C (mg/dl)
2.23±0.41^(++)^	2.90±0.50^(+)^	4.45±0.94^(^^*^^)^	5.23±1.94^(^^*^^)^	2.79±0.93	TG/ HDL

The data are offered as mean±SD *, **, and ***, respectively show significant differences at the levels of *P*<0.05, *P*<0.01, and *P*<0.001, between the healthy control and PCO groups. +, ++, and +++, showed significant differences between zinc-methionine-treated PCO groups and PCO control group at the levels of *P*<0.05, *P*<0.01, and *P*<0.001, respectively. TG: triglyceride; HDL-C: high-density lipoprotein cholesterol; Control: Healthy control group; PCO: Polycystic ovary control group; PCO-ZM 25, PCO-ZM 75 and PCO-ZM175 groups: polycystic groups treated with 25, 75, and 175 mg zinc-methionine, respectively

**Figure 1:**
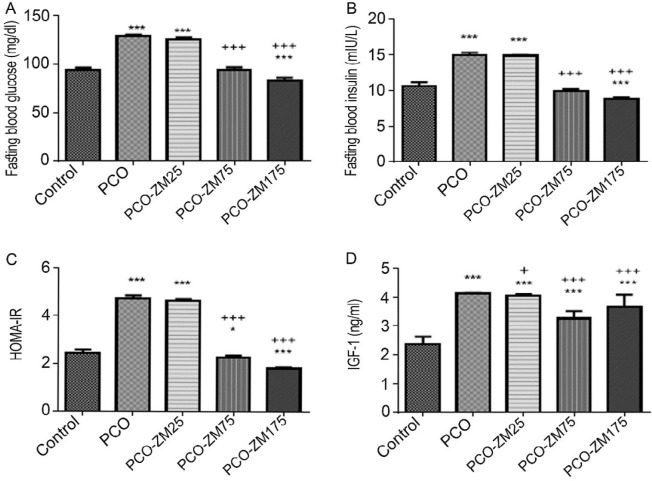
The effects of different levels of zinc-methionine supplementation on blood glucose (A), insulin (B), HOMA IR (C), and IGF1 (D). The data are presented as mean SD *, **, and ***, respectively show significant differences at the levels of *P*<0.05, *P*<0.01, and *P*<0.001, between the healthy control group and PCO groups. +, ++, and +++ showed significant differences between PCO treated groups with zinc-methionine and PCO control group at *P*<0.05, *P*<0.01, and *P*<0.001, respectively. Control: Healthy control group; PCO: Polycystic ovary control group; PCO-ZM 25, PCO-ZM 75, and PCO-ZM175 groups: polycystic groups treated with 25, 75, and 175 mg zinc-methionine; respectively

**Figure 2:**
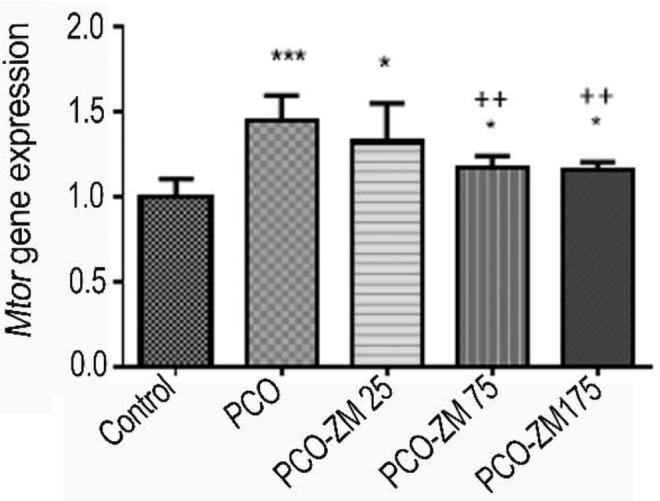
The effects of zinc-methionine supplementation on mTOR gene expression. The values are presented as mean±SD *, **, and ***, respectively show significant differences at the levels of *P*<0.05, *P*<0.01, and *P*<0.001, between the healthy control and PCO groups. +, ++, and +++ showed significant differences between PCO treatment groups with zinc-methionine and PCO control group at the levels of *P*<0.05, *P*<0.01, and *P*<0.001, respectively. Control: Healthy control group; PCO: Polycystic ovary control group; PCO-ZM 25, PCO-ZM 75, and PCO-ZM175 groups: polycystic groups treated with 25, 75, and 175 mg zinc-methionine; respectively

**Figure 3:**
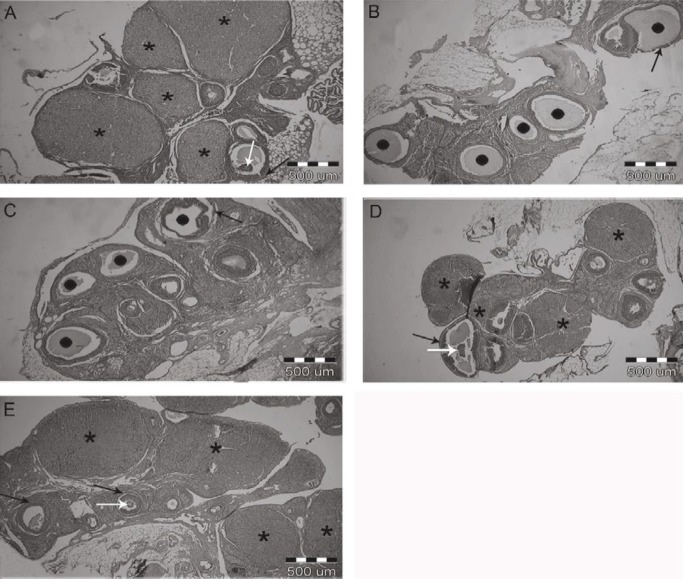
Effects of zinc-methionine supplementation on the evaluation of ovarian histology in different groups, arrows indicate layers of granulosa and oocytes. Scale bar, 500 µm. (A) Healthy control ovary, (B) polycystic control ovary, (C) ZM25-treated PCOS rat ovary. (D) ZM75-treated PCOS rat ovary, (E) ZM175-treated PCOS rat ovary. •, Follicular cyst; *, Corpus Luteum; Black arrows, Marker of granulosa cell; White arrows, marker of oocyte

## Materials and Methods

***Chemicals and Reagents***

Zinc-methionine (ZM) and estradiol valerate (EV) were purchased from Zinpro Corporation, (Eden Prairie, MN 55344 USA) and (Aburaihan Co. Tehran, Iran), respectively. Serum concentrations of gonadotropins including; luteinizing hormone (LH), follicle-stimulating hormone (FSH), 17β-estradiol (E2), progesterone (P4), total testosterone (T), and insulin were measured using enzyme-linked immunosorbent assay (ELISA) and IGF1 using sandwich (ELISA) and rat kits (Cloud-Clone Corp., Houston, TX, USA). Blood glucose was determined using an Autoanalyser kit (ERBA Diagnostic Mannheim GmbH, Mannheim, Germany). Triglycerides (TG) and high-density lipoprotein cholesterol (HDL-C) were evaluated enzymatically using kits (Pars Azmoon Inc., Tehran, Iran). The Tripure total RNA extraction Reagent (Roche, Switzerland) and cDNA synthesis kit (AccuPower® RT PreMix) were purchased from (Bioneer Corporation. Daejeon, South Korea). SYBR Green Real-Time PCR Master Mix High Rox was bought from Pars Tous (Pars Tous Co. Tehran, Iran). The *mTOR *and *Actb* primers were obtained from (Tag Copenhagen A/S, Denmark).

***Animal***

Animal care was conducted in accordance with the Animal Research Committee Guidelines of Islamic Azad University, Science and Research Branch based on criteria outlined in the Guide for the Care and Use of Laboratory Animals of the National Institutes of Health (NIH Publication No. 85-23, revised 1996). Experimental protocols were performed with the permission of the Institutional Ethics Committee of Islamic Azad University, Science and Research Branch.

In this study, virgin female rats (*Rattus norvegicus*. Sprague Dawley; SD), having three consecutive regular periods of the estrous cycle and weighing 145±5 g, were obtained from the Pasteur Institute of Tehran, Iran. The animals were housed in polycarbonate cages with paddy husk bedding, at a temperature of 22±3^ °^C and a 12 hr light (6:00-18:00) /12 hr dark (18:00-6:00) cycle. Animals had access to water *ad libitum* and were fed normal diets. A 2-week compromise was considered for animals. The duration of experiment was 57 days; (14 days of compromise, 28 days of induced PCO, and 15 days ZM-treated). In this study, we used ZM supplementation (Zinpro 120) from Zinpro Corporation, USA, which contained 12% pure zinc. Consequently, in order to provide the levels of 25, 75, and 175 mg/kg BW pure zinc, we used 208, 625, and 1458 mg/kg BW of Zinpro 120, respectively. 

Signs of Zinc Toxicity: Zinc is often considered to be relatively nontoxic, however, Acute toxicity estimate (ATE) for this mixture: LD_50_ (oral)= 5,250 mg/kg BW and acute toxicity data for metal constituents, LD_50_ (oral, rat): Zinc 630 mg/kg BW was determined as the concentration of LD_50_.

***Experimental treatments***

The study consisted of 45 female SD rats that after adaptation period were weighed and equally divided into five control and treated groups (n=9 per group) designated as:

A: Control groups 

1) Healthy rats that received the standard diet and were daily injected 0.4 ml of sesame oil intraperitoneally (Control).

2) Rats as PCOS controls that were injected subcutaneously with estradiol valerate (Aburaihan, Iran) (4 mg EV in 0.4 ml sesame oil/rat) for four weeks (PCO).

B: treated groups

1) PCOS rats that daily received 25 mg/kg BW pure zinc in the form of ZM by gavage (PCO-ZM25).

2) PCOS rats that daily received 75 mg/kg BW pure zinc in the form of ZM by gavage (PCO-ZM75).

3) PCOS rats that daily received 175 mg/kg BW pure zinc in the form of ZM by gavage (PCO-ZM175).

In the present study, PCOS was induced by EV due to the reported activities (21). Studies showed that administration of EV to adult rats leads to anovulation and cystic ovarian morphology (22). To ensure induction of PCOS, four rats from PCOS groups were sacriﬁced at the end of the injection period, and blood was collected by cardiac puncture, then hormonal studies and morphological were investigated. After verifying the induction of PCOS, treated groups received three levels of the organic supplement of ZM dissolved in distilled water and applied by gavage for 15 days. In order to prevent the interference effect of carrier, after the calculated Zinpro values, the amounts of required ZM were dissolved in distilled water to separate the carrier from ZM. After carrier deposition, the supernatant containing soluble ZM was gavaged to the rats.

***Biochemical and histological studies***

In order to measure biochemical parameters, after 15 days of exposure to treatments, animals were fasted overnight, weighed by digital scales, ketamine anesthetized, and blood samples (5 mL) were taken by cardiac puncture. To separate the sera, blood samples were allowed to clot and then centrifuged. Serum concentrations of FSH, LH, estradiol, progesterone, total testosterone, IGF-1, and fasting insulin (FINS) were determined by using specific commercial ELISA kits. The intra- and inter-assay coefficients of variation (CV) were <10 and <12% for all hormones. Fasting blood glucose (FBG) was estimated by an autoanalyzer; TG and HDL-C by an enzymatic colorimetric method and commercial assay kits as instructed by the manufacturer, and the intra- and inter-assay CV of all assays were <5%. To evaluate insulin resistance, homeostasis model assessment of insulin resistance (HOMA-IR) was calculated from the following formula:

HOMA-IR=FBG (mg/dL) ×FINS (mIU/l)/405. 

In the PCOS model, HOMA-IR> 2.8 was considered as insulin resistance [1]. To survey the effects of treatment on the reproductive organs, the ovaries and uterus were removed, stripped of fat and connective tissue, and weighed by a scale with an accuracy of ± 0 001 g. Then based on histological stages, the left ovary of each animal was fixed in 10% neutral buffered formalin. Samples were dehydrated (by alcohol solutions), cleared (by xylene), and embedded in paraffin blocks. Then blocks were cut into 5 μm thickness serially using a microtome and were mounted on gelatin-coated slides and deparafﬁnized in xylene and rehydrated by descending graded alcohol. Finally, for histological analysis, sections were stained with hematoxylin and eosin and were observed under a Zeiss Axioskop 2 microscope (Zeiss Fluorescent Microsystems, Gottingen, Germany).

***Quantitative real-time PCR analysis***

The right ovary of each rat was removed and immediately placed in RNA*later* (QIAGEN GmbH, Germany). Total RNA was extracted from the whole ovaries by using Tripure isolation reagent (Roche, Switzerland) according to the manufacturer’s protocol. Followed by RNA extraction, purity and concentration of RNA were measured by reading light absorbance at wavelengths of 260 and 280 nm using a spectrophotometer (Epoch microplate spectrophotometer biotek instruments inc. USA) and then samples were frozen at -70 °C. To synthesize cDNA, the isolated RNA of each sample (2 μg) was reversed using the cDNA synthesis kit (BIONEER, South Korea) according to the manufacturer’s instructions and was prepared within a ﬁnal reaction volume of 20 μl. Primer sequences for *mTOR* and *Actb* as a reference gene are shown in Table 1. Meanwhile, the mRNA expression reaction was performed using the Real-Time PCR system (ABI 7300; Applied Biosystems 7300 Real-Time PCR System, USA) and 2× SYBR Green qPCR reaction mix (Pars Tous, Iran), multiplied each of the genes desired, and the CT values were determined for each sample. The PCR process temperature program was as follows; the initial denaturation was run at 95 °C for 5 min, 1 cycle. Subsequently, 45 cycles of denaturation were performed in 1 min, annealing at 56 °C for 15 sec, and extension at 72 °C for 45 sec. All samples were run in triplicate, and each reaction contained a non-template control. The results for target gene (*mTOR*) were expressed as the amount relative average CT values of reference gene (*Actb*). The 2^–ΔΔCT^ method was used in relative gene expression.

***Statistical analysis***

Data were analyzed using SPSS Statistical Software, version 21. One-way ANOVA and *LSD* were used to determine statistically significant differences between the treatments (*P*<0.05). PCO groups were compared with the healthy control group and comparison was made between treated PCO groups and PCO control group. The results were shown as mean ± standard deviation and GraphPad Prism Software version 5.0a (Graphpad Prism, San Diego, CA, USA) was used to plot the graphs. A significance level below 5% was considered statistically significant.

## Results

***Weight of body and reproductive tissues***

In this study, the effects of ZM on PCOS were investigated by induction of disease in female SD rats. The results indicated that bodyweight in the PCO group was significantly increased compared to the healthy control group (*P*<0.01, Table 2). However, treatment with 75 and 175 mg/kg Zn decreased the body weight in the induced groups (*P*<0.01), and it was found that 75 mg/kg group had lower bodyweight compared with 175 mg group. The Ovaries quotiety was reported as ovaries weight/bodyweight (mg / 100 g). The ovaries quotiety was significantly greater in the PCO group compared with the control group (0.067±0.002 vs 0.055±0.005). Oral dosing (gavages) 75 and 175 mg/kg ZM significantly decreased ovaries quotiety compared to the PCO group. However, injection of EV showed a significant decrease in uterine quotiety in the PCO group compared to the control group (*P*<0.001). The doses of 75 and 175 mg/kg of ZM significantly inhibited the decrease in uterine weight in these groups (*P*<0.001).

***Reproductive hormones***

The induction of PCOS by EV increased the serum levels of LH, testosterone, and estradiol in the PCO group while decreasing the level of FSH and progesterone in comparison with healthy controls (*P*<0.001, Table 3). Oral 75 and 175 mg/kg gavages of ZM supplement increased FSH and progesterone levels compared with the PCO group and decreased the level of testosterone and estradiol (*P*<0.01).Treatment with 25 mg/kg ZM showed no significant effect on the mentioned parameters compared to PCO.

***Lipid profile***

In this study, EV treatment decreased HDL and increased both triglyceride levels and triglyceride to HDL ratio as compared to the healthy control group (Table 4). Zinc may also have a protective role in preventing atherogenesis obesity (23). Hence a significant decrease in TG levels and HDL/TG ratio was observed in treatments of 75 and 175 in comparison with the PCO group while those of HDL levels decreased. Thus these treatments could restore lipid parameters close to those in the control group. There was no significant difference between these groups compared to those in control.

***Insulin resistance***

Comparison of control and PCO treatments showed that induction of PCOS significantly increased levels of glucose (94. 2.19±2.19 vs 129.00±129.5), insulin (10.58±0.60 vs 14.96±036), HOMA IR (2.46±0.11 vs 4.73±0.10), and insulin-like growth factor-1(IGF1) (2.35±0.27 vs 4.12±0.02) (Figure 1, *P*<0.001). However, treatments of 75 and 175 mg/kg ZM showed lower levels of glucose, insulin, HOMA IR, and IGF1 compared with the PCO group. Interestingly, glucose (83.40±2.70), insulin (8.83±0.21), and HOMA IR (1.81±0.05) levels were lower in 175 mg/kg treatment compared with those in control. There was no significant difference between PCO and 25 mg ZM for glucose, insulin, and HOMA IR levels (*P*>0.05). Only, in the 25 mg group the IGF1 level was lower compared with the PCO group.

***Gene expression of mTOR***

*mTOR* gene expression in ovarian tissue was evaluated, and results showed that EV, which induced PCOS, significantly increased *mTOR* gene expression in the PCO group compared with the control group (1.45 ± 0.14 vs 1.00 ±0.10), (*P*<0.001). It was found that *mTOR* mRNA down-regulated in the PCOS treated groups with 75 (1.17±0.07) and 175 (1.16±0.04) mg/kg ZM levels compared with the PCOS group (*P*<0.01). Also, no significant difference was observed between the PCOS group treated with 25 mg/kg ZM and PCOS for gene expression (Figure 2,* P*> 0.05).

***Morphological evaluation***

Morphology of ovaries was compared in different groups to observe the therapeutic effects of ZM supplementation on histological characteristics (Figure 3). The ovaries in the healthy control group showed normal histology, large number of corpus luteum, and several follicles in various stages of development (Figure 3A). 

Therefore, observation confirmed the creation of the cyst and stopping of follicular development after 28 days of treatment with EV, due to lack of normal ovulation, cyst formation, and normal follicular growth distribution (21, 24). We detected unhealthy morphology with a large number of cysts with thin layer granulosa cells in the ovaries of the PCO group and also degenerated oocytes in the follicle. The absence of corpus luteum in this group suggested a complete induction of PCOS phenotype after 28 days of EV injection (Figure 3B). The ovaries in the 25 mg/kg ZM group exhibited no atretic follicles or corpus luteum while several cysts were present (Figure 3C). In PCOS groups treated with 75 and 175 mg ZM, ovarian morphology showed a lowered number of cysts and increased number of antral follicles and corpus luteum compared to the PCO group (Figures 3D, E). The ovaries in these two groups showed follicles in various stages with increased granulosa cells. Several corpus luteum and antral follicles indicated ovulation in 75 and 175 mg/kg ZM groups. The numbers of corpus luteum and antral follicle were greater in the 175 mg/kg ZM group compared with those of 75 mg/kg group, but the size was smaller.

## Discussion

Similar to this study, other studies have also shown that EV for four weeks increased the body and ovaries’ weight (21, 25). Obesity is one of the usual disorders in individuals with PCOS. Researchers have shown that weight gain in patients PCOS was due to increased visceral adipose tissue (26)**.** Studies have shown that IR and androgens have a synergistic effect on increased body fat distribution in individuals with PCOS and also showed that metabolic and endocrinological disorders are the most effective factors in increasing body mass index in women with PCOS (27). Obesity is related to increased oxidative stress that results in IR, so obese people usually have IR (28). However, in this study, 75 and 175 mg/kg ZM groups had a HOMA-IR <2.8, which indicated lack of insulin resistance and low oxidative stress. Zn is known as a member of the antioxidant system, and studies on organism and laboratory have shown that Zn deficiency increased oxidative stress (29, 30). ZM appears to decrease the oxidative stress and, consequently, IR through the antioxidant system and thereby improved the body and ovaries’ weight in 75 and 175 mg/kg ZM groups. However, same levels of ZM supplementation (75 and 175 mg/kg) increased percentage of uterine weight (ratio uterus weight/body weight*100), which was decreased in the PCO group. Unfortunately, studies on uterine weight and effect of IR on uterine weight in PCOS are limited, and some studies classified uterus as a target tissue for IR (31). Studies showed that insulin resistance and hyperandrogenism, common symptoms of PCOS, lead to change of uterine morphology, cell phenotype, and function of uterine cells (32). In this study, PCOS reduced the weight of the uterus which requires more investigation. No difference between PCO-ZM25 and PCO groups influenced the mentioned parameters, which can be due to insufficient Zn to improve the above parameters of this group.

In this study, the acyclic and intermittent production of extra estrogen may lead to positive feedback on LH release and negative feedback on FSH secretion which increases the ratio of LH/FSH in the blood. This alteration in the LH to FSH ratio increased the release of LH and decreased the amount of FSH in the PCO group (33). Studies showed that the main reason for enhanced LH concentration is abnormal positive feedback on LH secretion by mediating estradiol and negative feedback action of progesterone (34). Insulin resistance may also increase the release of steroids and LH through hyperinsulinemia (5). Increased estradiol level may be due to the peripheral conversion of androgens to estrogens, especially in adipose tissues. As noted, weight gain in the PCO group due to increased visceral adipose tissue and rate of more fat tissues may have resulted in further conversion and increased estradiol levels. Production of progesterone and androgens in ovarian theca-interstitial cells is initially controlled by the LH hormone. Since this hormone is high in the PCO group then LH is responsible for steroidogenic activity of theca cells (35). In letrozole induced PCOS, progesterone and LH levels decreased and increased in a dose-dependent manner, respectively. The amount of FSH in the low dose group tended to decrease, and excessive levels of testosterone indicated the aggregation of androgens, as it prevents the conversion of androgenic substrates to estrogen (36). In the PCO group, increased level of LH relative to FSH led to gradual increasing synthesis of androgens from ovaries. The serum progesterone level was low in the PCO group, which is in agreement with a previous study (37). Lower production of progesterone in the PCO group also indicates ovulation failure and low number of corpus luteum in this group (38). Supplementation at levels of 75 and 175 mg/kg, successfully restored progesterone levels to normal, which can be due to ovulation in PCO groups after receiving ZM. In the PCO group many follicular cysts without corpus luteum were observed (Figure 3). However, oral gavage of 75 and 175 mg/kg supplement, increased and decreased the FSH and LH levels, respectively, compared to the PCO control group. It has been reported that zinc deficiency in females leads to disorders in synthesis or secretion of FSH and LH (39). A precise mechanism for zinc action, especially in female sex has not been reported yet. However, a study on smokers showed that zinc supplementation significantly increased the level of FSH and LH by improvement of the gonadal activity and steroid feedback (40). ZM is also able to regulate the serum level of testosterone through the inhibition of aromatase enzyme (41). In relation to testosterone levels, studies have shown that individuals with normal zinc levels have a higher concentration of testosterone (42). A positive correlation between the plasma testosterone level and the concentration of sperm zinc has been reported, too (43). Murarka *et al, *in their study on male rats suggested that zinc deficiency reduced the activity of angiotensin-converting enzyme activity, which in turn reduced the level of testosterone (44). In pathophysiological conditions, it seems that Zinc performance on reproductive hormones is changing and its mechanism needs to be further investigated (44). Another explanation might be that insulin resistance increases steroids and it is likely that Zn decreases the levels of these hormones by reducing IR (Figure 1). Insulin reduces the production of the sex hormone binding globulin or hepatic SHBG and therefore increases the levels of free androgens (9). Therefore, it seems that reducing insulin in 75 and 175 mg Zn groups is a factor in decreasing LH, testosterone, and estradiol levels.

One of the consequences of PCOS is dyslipidemia and imbalance in lipid profiles due to hyperandrogenemia (45). On the other hand, studies have shown that zinc supplementation has beneficial effects on plasma lipid parameters and significantly reduces total cholesterol, LDL-C, and TG. In addition, it has significantly increased HDL in healthy individuals and patients and is able to reduce the potential for atherosclerosis and associated illnesses (23). Insulin resistance has also been reported to reduce levels of antioxidant activity (46). According to the findings, it can be said that ZM at high levels, may reduce TG levels and TG/HDL ratio via the antioxidant system. Zn as antioxidant performs in two ways; A) Protects proteins and enzymes that act against oxidation and ROS and B) Prevents the formation of ROS (47). In the present study, the 25 mg/kg ZM level was not sufficient to act as an antioxidant.

Studies have shown that insulin metabolic abnormalities in women with PCOS, increase hepatic gluconeogenesis and defects in insulin receptor signaling, which subsequently have a negative effect on the transfer of glucose via insulin (48). The proposed mechanism for increasing insulin in PCOS is that glucose remains in the blood for a long time and stimulates the pancreatic β-cells to release more insulin in order to improve muscle glucose consumption (7). Insulin, in turn, stimulates the production of androgens from the ovaries via activation of its homologous receptor in the PCOS individuals (9). On the other hand, elevated serum insulin levels will increase the bioavailability of IGF1 (49). Insulin, by regulating the synthesis of IGF-binding protein-1 (IGFBP-1) and sex hormone-binding globulin (SHBG) proteins, increases the IGF1 concentration in the liver (7). It has been reported that gradual increase in insulin level and IGF1 activity at puberty may be one of the factors for PCOS induction (50). Zn plays an important role in the production, storage and diffusion release of insulin, and in obese people, Zn concentration is low in plasma, erythrocytes, and serum (23). In addition, there is evidence of the Zn stimulatory effect on leptin production in adipose tissue. Probably the mechanism of Zn effect in reducing hyperglycemia in obese and/or diabetic animals is due to increased leptin expression induced by this mineral (51). 

In this study, 75 and 175 mg/kg ZM treatments dramatically reduced glucose levels, which was even lower in comparison to the healthy control group. Zn deficiency may lead to glucose intolerance and insulin resistance, diabetes and coronary artery disease (52). On the other hand, insulin is stored as zinc-insulin crystals in pancreatic β-cells, and Zn deficiency may be associated with insufficiency of insulin secretion (53). In the present study, application of Zn reduced insulin levels via interfering with insulin sensitivity of tissues. Studies have shown that zinc-2α-glycoprotein has reduced Insulin receptor substrate-1 (*Irs-1*) and glucose transporter 4 (*Glut4*) expressions in human adipocytes, which play important roles in insulin sensitivity of adipose tissues (54). So, ZM may also decrease the insulin level in PCOS mice with more adipose tissue by similar mechanisms, and it affects glucose intake, which can affect HOMA-IR levels. Leptin production would be affected by cytokines too (51). Thus, it is possible that increasing the production of cytokines in response to zinc supplements, with is the mechanism responsible for increasing the amount of leptin, leads to a decrease in blood glucose and insulin resistance in 75 and 175 mg/kg treatment groups. It has also been reported that nutrients activate *mTOR* and cause IR *in vitro*, so *mTOR* activation blocks insulin signaling and induces IR (55). It can be claimed that down-regulated *mTOR* expression in 75 and 175 mg/kg ZM groups (Figure 2) is the cause of insulin resistance reduction in these groups when compared to PCO. Imamoglu *et al.* reported positive effects of Zn on IGF-1 levels. These researchers showed the positive effects of Zn on IGF-1, increased endogenous growth hormone sensitivity, and physiological secretion of growth hormone (56). However, in the present study, increasing effects were observed in comparison with healthy controls, but decreasing effects were observed when compared to the PCO, and it may be due to association with insulin.

Increased *mTOR* gene expression in the PCO group compared to the healthy control group was due to the raised glucose, insulin, testosterone, and IGF-1 levels, which incremented the expression of the *mTOR* gene in ovaries (57). Estrogen has also been reported as one of the activators of *mTOR *(58). Researchers showed that *Rictor*, as an essential component of the *mTORC2* complex had a higher expression in PCOS mouse and also stated that it could play an important role in the development of PCOS (59). While Kim *et al.* reported that Zn increases *mTOR* gene expression in the *in vitro* model (60). We found that Zn supplementation reduced the expression of *mTOR* compared to PCO and 25 mg/kg groups, the mechanism of which is unknown. However, a combination of zinc and insulin may stimulate phosphorylation of protein in the *mTOR* pathway in skeletal muscle tissue and enhance *mTOR* signaling (19). Insulin resistance seems to enhance or weaken *mTOR* gene expression. Insulin resistance was declined in 75 and 175 mg/kg ZM levels. Therefore, the *mTOR* gene expression has also decreased in these groups.

Defective and disturbed steroidogenesis in theca cells is one of the reasons for the occurrence of PCO. There is evidence that hyperandrogenemia in mouse and rat models reduce oocyte numbers and increase degenerated oocyte and androgens roles in follicular growth and development through the effect on granulosa cells (61). Furthermore, increase in the apoptosis of granulosa cells in ovarian follicles has been reported in EV-induced PCOS (62). In fact, there is a high correlation between hormonal levels and ovarian morphology, and improved hormonal levels in 75 and 175 mg/kg ZM groups could be the reason for improved ovarian morphology in these groups as compared with the PCO group. Another reason can be related to the *mTOR* gene expression because it has an important role in controlling the ovarian follicle growth by regulating granulosa cell proliferation (63), which can be a reason for differing ovary morphology between groups.

## Conclusion

In general, the present study showed that ZM may be a useful treatment for improving PCOS. However, increasing zinc application, especially at 175 mg/kg BW, through reduction of body weight, LH, testosterone, estrogen, insulin resistance, and triglyceride levels, significantly helps to enhance ovulation in rats with PCOS. It can be argued that ZM reduces insulin resistance via down-regulation of the *mTOR* gene expression and results in reduced release of steroids and LH, which in turn correlate with other parameters in subjects with PCOS.
